# Application of Arterial Spin Labelling in the Assessment of Ocular Tissues

**DOI:** 10.1155/2016/6240504

**Published:** 2016-03-15

**Authors:** E. Vaghefi, B. Pontré

**Affiliations:** ^1^School of Optometry, University of Auckland, Auckland 1023, New Zealand; ^2^Department of Anatomy with Radiology, University of Auckland, Auckland 1023, New Zealand

## Abstract

Arterial spin labelling (ASL) is a noninvasive magnetic resonance imaging (MRI) modality, capable of measuring blood perfusion without the use of a contrast agent. While ASL implementation for imaging the brain and monitoring cerebral blood flow has been reviewed in depth, the technique is yet to be widely used for ocular tissue imaging. The human retina is a very thin but highly stratified structure and it is also situated close to the surface of the body which is not ideal for MR imaging. Hence, the application of MR imaging and ASL in particular has been very challenging for ocular tissues and retina. That is despite the fact that almost all of retinal pathologies are accompanied by blood perfusion irregularities. In this review article, we have focused on the technical aspects of the ASL and their implications for its optimum adaptation for retinal blood perfusion monitoring. Retinal blood perfusion has been assessed through qualitative or invasive quantitative methods but the prospect of imaging flow using ASL would increase monitoring and assessment of retinal pathologies. The review provides details of ASL application in human ocular blood flow assessment.

## 1. Ocular Blood Perfusion Quantification

**Figure 1:**
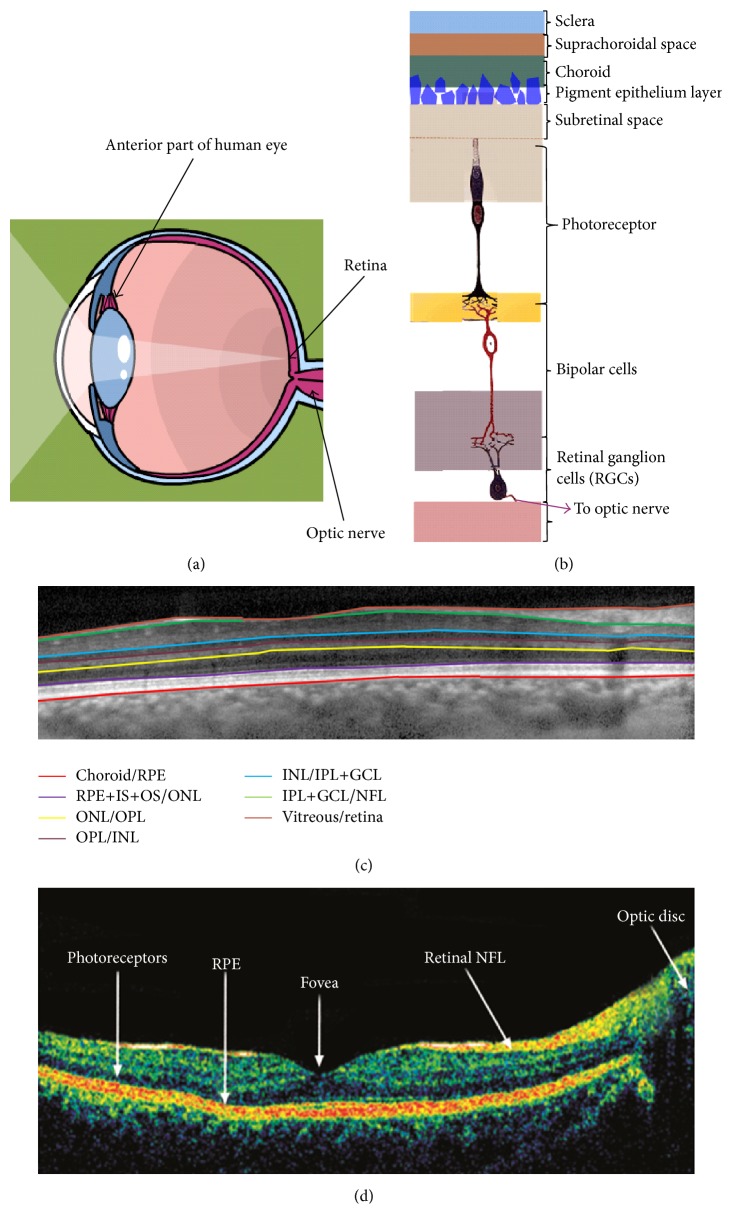
The layered structure of the retina. (a) General anatomy of the eye, including the retina lining the back of the eye; and (b) the cellular component of retinal stratified structure. (c) The segmented raw retinal OCT image overlaid with seven identified boundaries. (d) Raw OCT image of the retina.

Our sense of vision is critically dependent on all the components of our eye to function cohesively, so we will have a clear image of the outside world. The retina is the light-sensitive tissue that is lined on the inside surface of the eye and contains nerve cells, which convert incoming light into electrical impulses (Figure 1). Anatomically, retina is a highly stratified tissue, consisting of multiple cell types organized into its layered structure. Each layer of the retina performs distinct and yet interdependent functions, which in conclusion support the phototransduction process [1]. Briefly and moving from inside in contact with the vitreous outside, these are the inner limiting membrane, nerve fibre layer, ganglion cell layer, the inner plexiform layer, inner nuclear layer, the outer plexiform layer, outer nuclear layer, the external limiting membrane inner/outer segment of photoreceptors, and the retinal pigment epithelium. Like all the other tissues in the human body, the retinal layers of different cell types need oxygen, and hence blood supply, to survive.

The two major sources of blood supply to the mammalian retina are the retinal and the choroidal blood vessels [2]. The choroidal vascular bed receives the bigger blood flow of about 65–85% of the total blood flow and the remaining 15–35% flows to the retina through the central retinal artery [3]. While the choroidal circulation is vital for the maintenance of the outer retina and particularly the photoreceptors, the retinal vasculature nourishes the inner retinal layers. The arterial input to the eye is derived from the internal carotid artery and includes central retinal artery, the short and long posterior ciliary arteries, and the anterior ciliary arteries. Venous outflow from the eye is primarily via the vortex veins and the central retinal vein. The choroidal arteries arise from long and short posterior ciliary arteries and each of the posterior ciliary arteries breaks up into fan-shaped lobules of capillaries that supply localized regions of the choroid. The arteries pierce the sclera around the optic nerve and fan out to form the three vascular layers in the choroid: outer (most scleral), medial, and inner (nearest Bruch's membrane of the pigment epithelium) layers of blood vessels [4, 5]. Any deficiency of either of these two retinal circulations could result in blood perfusion alterations in the retina which is linked to several retinal pathologies [6].

Perfusion allows for the delivery of oxygen and nutrients to tissues by means of blood flow and it is one of the most fundamental physiological parameters [7]. Disorders correlated with blood perfusion such as stroke account for much of the medical morbidity in industrialized nations, and blood flow alterations also commonly accompany other pathophysiological changes such as cancer, epilepsy, and neurodegenerative diseases [8]. Hence, the measurements of perfusion have direct diagnostic value. Tissue perfusion is usually measured using a diffusible tracer that can be exchanged between the vascular compartment and tissue [9]. Conventional fluid flow describes the volume of liquid passing a given point per unit of time and has a unit of mL/min. However, perfusion flow is a more useful physiological measure. Perfusion flow is the volume of fluid (i.e., blood) passing in and out of a given weight of tissue per unit of time. That is because the cerebral and retinal blood flow should be distinguished as a rate for a specific tissue weight [10].

There are many methods of measuring blood perfusion, including methods based on computed tomography (CT), laser Doppler, single-photon emission computed tomography (SPECT), and MRI. The majority of these techniques require the injection of an exogenous tracer that acts to alter the signal intensity as the contrast agent moves through the blood, allowing the quantification of tissue perfusion. Both CT and SPECT techniques provide high sensitivity to perfusion [11] but are unsuitable for serial studies requiring multiple examinations owing to the use of ionising radiation. MRI techniques using gadolinium based contrast agents (GBCA) also provide high sensitivity while avoiding the use of ionising radiation. However, the risks associated with GBCA such as nephrogenic systemic fibrosis (NSF) make such techniques unsuitable in at-risk patients. The use of contrast agents that are not gadolinium based such as fluorinated halocarbons [12], deuterated water (2H_2_O) [13, 14], and 17O-water [14] has been investigated. The use of arterial spin labelling (ASL) techniques eliminates the requirement for externally administered agents and exploits the advantages offered by MRI-based techniques [15, 16].

## 2. ASL

The primary benefit of ASL over other MRI-based techniques is that the use of the blood itself as a tracer eliminates the risks associated with GCBA and other exogenous tracers. As a result, ASL is of most use in cases where the use of an exogenous contrast agent is contraindicated [17] or in cases where patients require multiple perfusion assessments in a single study [18]. In studies of the eye, for example, it may be appropriate to assess perfusion under a variety of conditions during the same study, making techniques based on exogenous contrast agents undesirable [19, 20].

ASL requires that the arterial blood supplying the tissues of interest is labelled by modifying its magnetization. Image contrast is affected by the presence of the labelled blood in the imaging volume. Acquiring an image of the tissues of interest after a sufficient delay time to allow the labelled blood to enter the imaging volume results in a decrease in signal related to the local tissue perfusion. Comparing the labelled images to those acquired without the labelled blood allows regional variations in tissue perfusion to be identified. Further, this signal can be modelled to determine the tissue perfusion at each voxel. In both the qualitative and the quantitative applications of ASL, the resulting signal depends on the tissue properties, timing considerations, and imaging parameters. For this reason, ASL techniques used in any study need to be specifically optimized for the tissue of interest [21, 22].

### 2.1. Applications of ASL

In a clinical setup, ASL can be grouped with other anatomical (e.g., spin-lattice T1 or spin-spin T2) or functional (e.g., fMRI) imaging sequences in order to provide a comprehensive assessment of the imaged organ [23]. To date, the major clinical applications of the ASL have been focused on studying the brain and its disorders [24]. Several studies have used ASL to detect regional hypoperfusion in patients suffering from Alzheimer's dementia ([25, page 2000], [26]) or frontotemporal dementia [27, 28]. Epilepsy is another neurological disorder in which ASL can be applied for diagnosis and management. Interictal hypoperfusion measured by ASL has been shown to correlate with interictal hypometabolism ([29, 30], [31, page 200]). ASL has also been used in conjunction with other modalities to monitor the diagnosis and treatment effects of several brain affective disorders such as depression [30, 32], schizophrenia [33], Parkinson [34], and hypoperfusion of prefrontal cortex [35]. Furthermore and combined with fMRI, ASL is proved to be an appealing approach for imaging brain activations during long time scale processes and more ecological paradigms such as motor learning [36], emotion or mental states [37–39], mood changes [40, 41], and natural vision [42]. The utility of ASL in detection of migraine [43, 44] and focal seizure [44] has also been demonstrated in several case reports. ASL can be combined with fMRI to look at brain's oxygenation and functionality since it provides absolute quantification of brain blood perfusion and is less susceptible to baseline drift and motion artefact [45, 46]. Finally, since ASL is reagent-free it has become an appealing technique in paediatric studies, as a biomarker for functional brain development in both healthy populations and developmental disorders [47, 48].

Apart from the field of brain research, new applications of ASL are being investigated around the world. Preliminary studies of the applicability of ASL in the cardiovascular investigations [49] and its related pathologies such as stroke have been looked at [50, 51]. ASL has also been obtained from postischemic extremities in patients with peripheral vascular disease [49]. The kidney is another highly perfused tissue where ASL has also been used [52]. Lastly, since tumorous tissue usually has higher perfusion than healthy tissue, ASL has been applied for detection and grading of tumours [53, 54]. One reason for the spread of ASL's clinical usage has been the verification of its measurements against previously stablished flowmetry methods.

ASL MRI has been validated against other quantitative technologies such as Dynamic Susceptibility-Weighted Contrast-Enhanced MRI [55–57], PET scan, and using different exogenous contrast agents [58, 59]. Furthermore, since ASL image contrast is not based on susceptibility effects, it could be used to study regions of high static field inhomogeneity [60–62]. This property of ASL is especially valuable while imaging highly layered structures such as the eye's retina [63, 64].

ASL implementations are now commercially available on all major MRI platforms and their reproducibility has been confirmed by several multicentre studies [46, 65]. However, before applying this technique in the clinic, many of its technical parameters have to be studied and optimized. Here we are going to review the effects of a number of these factors on the quality of the ASL measurements.

### 2.2. Technical Considerations

Early implementations of ASL used a series of short labelling pulses (PASL), to label the arterial blood [66–68]. The shorter pulses mitigate the SAR issues and high power deposition, but the nonadiabatic nature of the labelling pulses results in decreased labelling efficiency and lower ASL signal.

The next generation of ASL implementations is known as continuous ASL (CASL) [16]. In CASL schemes, the blood that passes through the labelling slab is continuously affected and allows blood magnetization to reach a steady-state, maximising the signal difference between the labelled and control conditions [69]. However, this labelling scheme leads to increased magnetization transfer effects that would decrease the accuracy of the measurements. The long labelling pulses used in CASL are particularly problematic in higher field strength systems. At these higher fields, specific absorption rate (SAR) is increased, leading to higher amounts of RF energy being absorbed and potentially increasing local heating in tissues. Longer RF pulses, like those used in CASL, also lead to increased SAR, exacerbating any potential heating occurring in patients [44, 70].

**Figure 2:**
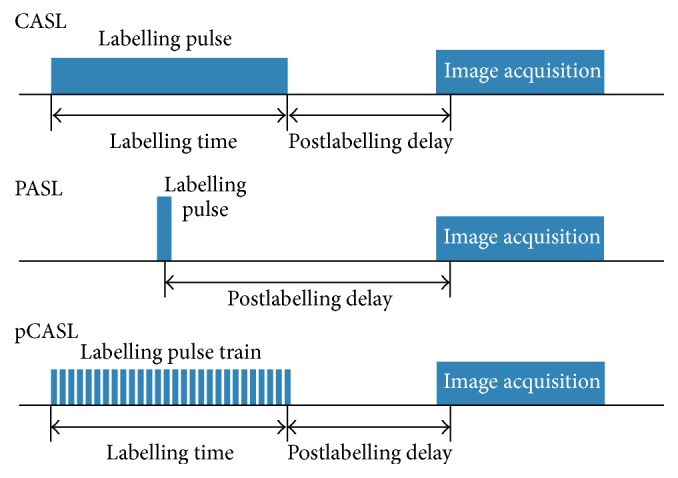
Different implementations of ASL pulse sequence for MR, continuous (CASL), pulsed (PASL), and pseudo-continuous (pCASL) technique.

The latest implementation of ASL in many modern applications is the pseudo-continuous ASL (pCASL) (Figure 2), which is used to overcome the limitations of PASL and CASL [15, 60]. Rather than using a single continuous labelling pulse like CASL, 1000 or more shaped magnetization pulses are very rapidly applied to label the arterial blood. Compared to CASL, pCASL provides superior labelling efficiency and is compatible with modern body coil RF transmission hardware that is now ubiquitous on clinical MRI scanners. In practice, the signal-to-noise ratio (SNR) of the pCASL implementation is higher than other methods due to two reasons. Firstly, in pCASL implementation the temporal duration of the labelled slab is longer which in turn leads to larger volume of labelled blood that is delivered to the tissue, leading to an increased SNR. Secondly, even for an interval of equal temporal duration, it has been shown that the labelled magnetization delivered to the blood is higher using pCASL [71].

Over the past decade, there has been a general trend to the use of higher field strength systems owing to the higher overall SNR. Theoretically, the expected ASL signal will increase proportional to the strength of the main magnetic field. Wang et al. [69] studied the effects of the magnet field strength on the implementation of ASL to quantify the cerebral blood flow. They reported that, using similar pulse sequences, a 4 T magnet generated more than twice the amount of ASL signal compared to a 1.5 T magnet, consistent with the theoretical expectations. Other studies have used 7 T magnet strength to apply ASL to human brain [72, 73]; however, it has been noted that since these high strengths are much more susceptible to off-resonance fields especially at the tagging location, a robust prescan procedure is needed to optimize the ASL parameters [74].

In all forms of ASL, ideally the labelling pulse will result in perfect and complete labelling of the arterial blood. In this ideal case, all of the blood passing through the imaging volume will contribute to the ASL signal. However, in magnets with clinical field strengths, the lifetime of the magnetically tagged blood is about 1300–1750 ms. The labelling lifetime along with the timing delays used in the pulse sequence will influence labelling efficiency. The postlabelling delay time (the delay between the labelling pulse train and acquisition) needs to be set so that the labelled blood arrives at the tissue of interest when it is acquired. Shorter delays can be used to reduce scan time but come at the expense of potentially inefficient labelling since the labelled blood has not had sufficient time to arrive at the imaging slice. A longer delay could be used to ensure that more labelled blood perfuses the tissues of interest but labelling efficiency may be reduced owing to T1 relaxation effects [75].

Aside from timing and protocol-specific effects, labelling efficiencies in ASL also depend on the hardware used. Higher magnetic field strength systems provide increased efficiency in ASL studies [76] owing to the increase in overall SNR as well as the lengthening of T1 in tissues [70]. The multichannel head coils have been shown to increase the SNR of the ASL scans [77] and allow for the use of parallel imaging techniques [45]. The coil arrays used in parallel imaging can be exploited to decrease the echo time (TE) and maximise the signal from tissues with very short T2, such as the retina [78]. The higher SNR achieved by using multichannel coils has also been shown to provide more accurate quantification of tissue perfusion [79–81]. The gain in SNR is also mentioned when surface coils are used in conjunction with standard head coils, imaging the brain, the kidney, and the eye [82].

Similar to other MRI techniques, ASL is also sensitive to motion and susceptibility artefacts. Motion artefacts are an issue in all MR imaging techniques owing to the effectively long scan times, resulting in difficulties for some patients to remain still throughout the examination. In ASL, misregistration resulting from motion between the control and labelled data can cause local or global changes in ASL signal not related to tissue perfusion [83–85]. Magnetic field inhomogeneities are problematic in EPI-based protocols such as ASL where the long echo trains used can result in image distortions or significant signal loss. Field inhomogeneities will also affect the labelling efficiency, particularly in pCASL techniques owing to the nonadiabatic nature of the labelling pulses [22]. Artefacts specific to ASL studies, such as enhanced T1 shortening owing to the presence of gadolinium based contrast agents, and insufficient suppression of intravascular spins can also result in suboptimal image quality [83–85].

Fortunately, many of these issues can be partially mitigated by the use of background suppression [86, 87]. Blood labelling in ASL is inherently inefficient with only 1-2% of the spins in the blood contributing to the signal, so the use of effective background suppression will increase ASL signal-to-noise ratio and sensitivity and result in improved quantification of perfusion measures. In order to incorporate background suppression in ASL implementation, an initial saturation pulse is applied to the imaging region. This saturation pulse is then followed by carefully timed magnetization inversion pulses. Such combination results in the longitudinal magnetization of the static target tissue being close to zero at the time of image acquisition. Meanwhile, the tagged arterial blood water molecules that are passing through the target tissue have not experienced the initial saturation pulse, but only the inversion pulses [88–90]. Hence, the ASL perfusion signal is preserved, while the static tissue signal is nearly eliminated.

### 2.3. Applicability of ASL in the Eye

The mammalian retina is found to have a high rate of perfusion [91–94], which is required for the maintenance of the physiological homeostasis in the highly compartmented ultrastructure of the retina. Retina is the most metabolically active tissue in the human body [95] and is highly sensitive to the local perfusion rates of the retinal and choroidal circulations. The choroidal arteries, which feed the outer retina, arise from the posterior ciliary arteries that penetrate the sclera around the optic nerve [4]. Simultaneously, the retinal vasculature network is branched from the central retina artery and feeds the deeper layers of the retina. The central retina artery itself receives its blood supply from the Circle of Willis, which is formed by an arterial polygon and supplies the blood of the eye and the brain.

**Figure 3:**
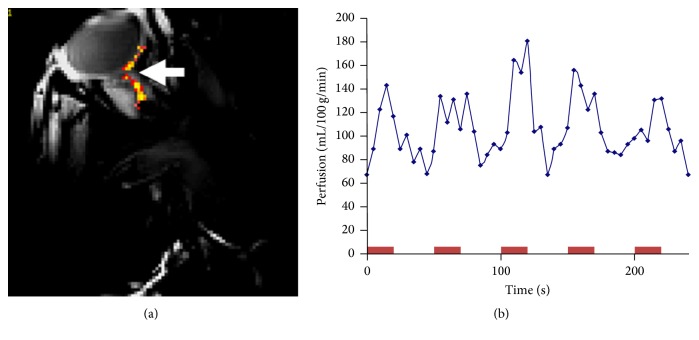
(a) Statistical map extracted from our ASL dataset is showing the correlation map of voxels (arrow) activated to the visual stimulus (*Z* > 3.23). (b) ASL perfusion signal in mL/100 g/min units is extracted from active voxels shown on (a) over the time course of our experiment. Red blocks denote the periods of light stimulation.

The retinal circulation has no autonomic innervation and is regulated by local factors [6] while the choroidal circulation is not autoregulated and is mainly controlled by sympathetic innervation [96]. Using a 9 T Phillips magnet in a preliminary study, we have previously shown that ASL can be applied to image this nonregulated blood flow in the retina (Figure 3). The perfusion rates of the blood in retina have been tried to be measured using other methods, including laser speckle [97], ultrasound combined with enhanced depth imaging OCT [98], high frequency immersion ultrasound [99], OCT Angiography [100, 101], Doppler OCT [102], ultrahigh speed swept source/Fourier domain OCT [103], and laser Doppler flowmetry [104]. Most of these techniques have not been transformed to the clinical setup, due to their limitations in sensitivity, reproducibility, and accuracy. However, the perfusion rates of the retinal and choroidal circulations are of clinical importance as they are altered and affected in many of the common retinal pathologies.

Several studies have shown that, within retina, the outer segments of the photoreceptors are the most metabolically active [105, 106]. Due to such high demand of blood perfusion, the ability to regulate steady blood flow in the retina is essential for the health of this tissue. Normal blood flow within the retina and choroidal vasculatures is altered in a number of retinal disorders that affect the overall vision.

Diabetic retinopathy (DR) is an ocular manifestation of diabetes which disturbs the normal retinal vasculature function and often leads to capillary occlusion and if left untreated to vascular proliferation [107, 108]. By the time that the manifestation of DR becomes clinically observable, irreversible changes to the retinal vasculature network have already occurred. These changes (i.e., proliferation of the retinal vasculature) are in response to the tissue hypoxia which is known to occur in DR; however, the exact time when hypoxia begins is as yet unknown [109]. In ophthalmic clinical studies, it has been shown that retinal blood flow is reduced before and in the early stages of DR [110–112]. This initial flow reduction is then followed by an increase in retinal blood flow, possibly due to the release of vascular endothelial growth factor (VEGF), which in turn leads to proliferative retinopathy [113, 114].

Glaucoma is a term encompassing a group of disorders of the eye which generally damage the optic nerve. Often the onset and progression of glaucoma are accompanied by an increase in the intraocular pressure (IOP) of the patient. There is evidence that there is a relationship between ocular perfusion and damage progression in patients with glaucoma [115]. There is further evidence that an eye with elevated IOP could experience conditions similar to those suffering from reduced retinal perfusion [105, 116]. Due to the effective autoregulation in the retinal circulation, the decreased perfusion pressure of the eye suffering from glaucoma does not affect the PO_2_ of the inner retina [117]. On the other hand, the choroidal blood flow was shown to be decreased in glaucoma, resulting in hypoxic conditions at the photoreceptors [116].

Retinopathy of prematurity (ROP) is affecting premature infants and it is marked by abnormal growth of blood vessel in the retina. ROP progresses firstly by delayed retinal vascular growth after birth and partial regression of existing vessels. This is then followed by neovascularization induced by hypoxia [118]. ROP was first associated with exposure to high levels of oxygen after the initial description of the disease [119]. In order to maintain healthy and sufficient blood perfusion levels in premature infants, currently supplemental oxygen is closely monitored [120]. The current clinical treatment to stop the progression of this condition is the indirect laser photocoagulation to bring the perfusion rates of the retina towards normal levels [121].

Retinal detachment is a condition that could occur due to various often advanced pathologies of the retina. In this condition, the retinal tissue peels away from the RPE [122]. During retinal detachment progression, the photoreceptor segments are separated from their supporting vasculature. This process leads to lower oxygen values at the photoreceptors site, inducing local hypoxic conditions and increasing the cell death rate under this condition. It has been shown that retinal detachment is correlated by altered choroidal perfusion rates [123].

## 3. Application of ASL in Animal Models

The ASL modality is capable of noninvasively quantifying blood perfusion in scanned tissues. A recent study showed that ASL could provide sufficient resolution and SNR to accurately measure the different blood flow of the retinal and choroidal circulations in mice eyes [63]. Furthermore, the same study showed that ASL can correctly quantify the changes of blood flow modulated by anaesthetics in their animal model. It was mentioned that the retinal blood flow changed from 1.3 ± 0.44 to 0.88 ± 0.22 mL/g/min and choroidal blood flow changed from 7.7 ± 2.1 to 4.3 ± 1.9 mL/g/min due to anaesthesia.

In a following study, a mouse model of diabetic retinopathy was imaged with ASL [124]. The blood flows of the mice were quantified at early and late time points after onset of hyperglycemia. It has been reported that the choroidal blood flow was reduced by 20% in the diabetic group compared with the control group after 10 weeks. After 30 weeks, it was observed that both choroidal and retinal blood flows were notably lower in the mice model of diabetic retinopathy. The visual performance of these animals was also found to be significantly worsened.

In the third study from the same authors, changes in retinal and choroidal blood flows of the mouse model of retinitis pigmentosa were measured with ASL [125]. Here it was observed that the retinal blood flow was decreased consistently and significantly through the course of the study. The same effect was not observed in the choroidal blood flow though.

Reference [126] measured the retinal and cerebral blood flows in rats under light and dark adapted conditions using ASL. These measurements were then reconfirmed with fluorescent microspheres. The choroidal blood flow was measured at 64.8 ± 29 *μ*L/min during dark adaptation and 66.0 ± 17.8 *μ*L/min during light adaptation condition. Retinal BF was 11.6 ± 2.9 *μ*L/min during light adaptation and between 8.2 and 9.9 *μ*L/min under dark adapted environment. A 10 Hz flickering light stimulation was also applied which led to significantly higher retinal blood flow but not the choroidal blood flow 13.5 ± 3.2 *μ*L/min.

ASL was utilized to obtain high resolution blood flow measurements of rat models of retinitis pigmentosa [127]. A CASL pulse sequence and an 11.7 T small animal MRI were combined with customized surface coil to implement the ASL technique here. It was hence possible to have 44 *∗* 44 *μ*m in-plane resolution and differentiate between retinal and choroidal circulations in rats' retinas. This study found that choroidal and retinal circulations have different susceptibility to progressive retinal degeneration in this animal model.

A follow-up study looked at applying ASL to look at the effects of acute hypertension on the choroidal and retinal blood flows, in a rat model [128]. In this study, an autoregulatory behaviour was observed in the retinal blood circulation, while a baroregulation response was imaged in the choroid vasculature. The authors believe that their animal model was useful in studying retinal and choroidal vascular dysregulation.

The most human-like implementation of ASL in animal models was done using eight baboons and a 3-Tesla clinical scanner [129]. The retinal blood flow was measured under normal and hypercapnia conditions in these animals. It was found that base blood flow from the posterior retina was 83 ± 30 mL/100 g/min while in hypercapnia this was increased by 25 ± 9%.

The above-mentioned studies have established the applicability and accuracy of ASL blood in studying the models of different retinal conditions. Next, we are reviewing the application of the ASL to human tissue.

## 4. Application of ASL in Human Retinal Tissue

One of the first studies that have used a conventional standard and commercially available 3 T clinical magnet to investigate the feasibility of measuring blood flow of the human retina using ASL was done by [130]. Here, the blood flow measurements were obtained in five healthy individuals using an 8-channel array head coil. A CASL was used in this study and the labelling was placed 5 cm below the optic nerve to invert the magnetization of the blood in the carotid arteries below the Circle of Willis. The inverting pulse duration was varied between 500 and 2000 ms to optimize the sequence for achieving maximum SNR. Additionally, background suppression technique was also employed in order to improve the quality of the obtained results. The two main sources of image artefact in ophthalmic MRI are ocular motion artefact and partial volume effects [131, 132]. The former is due to involuntary eye movement and the latter is due to the proximity of the eye to the surface of the body. In the [130] study, these artefacts are minimized by usage of a fixation target and blink-synced image acquisition. The blood flow estimation was performed using a single-compartment model [133, 134]. Using this model and assuming labelling efficiency of 0.85, brain-blood water partition coefficient of 0.9 g/mL [135], and longitudinal relaxation rate of 0.67 s^−1^ for the retinal tissue [136, 137], the measured blood flow to a section of the retina around the fovea was measured at 1.75 ± 0.54 *μ*L/mm^2^/min.

A follow-up investigation tried to differentiate between the human retinal and choroidal blood flow, in a clinical setup [138]. Four healthy individuals were scanned by ASL under normocarbia (partial end-tidal pressure values = 40 mmHg) and hypercarbia (partial end-tidal pressure values = 50 mmHg) conditions. The MRI setup was similar to a previous study [130] but a pCASL pulse sequence was applied here. Using a similar single-compartment model and parameter assumption as before, the baseline blood flow was measured at 1.55 ± 0.17 *μ*L/mm^2^/min. This value increased during hypercarbia to 1.96 ± 0.18 *μ*L/mm^2^/min. Overall, hypercarbia caused a 26% relative increase of blood flow from the normal condition which is calculated to be 6.7% increase in the blood flow per 1 mmHg. This study, by controlling the breathing conditions, showed that when the clinical ASL does not have the spatial resolution to differentiate the retinal and choroidal blood flows, the obtained measurements are dominated by the latter vascular blood flow.

A similar but separate study was performed on 5 healthy subjects under normal and hypercapnia conditions [139]. Eye fixation and queued breathing techniques were employed to minimize the motion artefact. The ASL labelling plane was placed 7 cm below the optic nerve, and ASL background suppression was also employed. The background suppression pulse timing for ASL of the human retina is investigated in depth and optimized in a later publication [90]. Here it was measured that the group-averaged peak blood flow value was at 93 ± 31 mL/(100 mL min). Furthermore, profiles of blood flow from sclera to vitreous and across the thickness of the retina showed that under hypercapnia condition flows were increased from 93 ± 31 to 104 ± 35 mL/(100 mL min).

One study investigated the levels of blood perfusion in retina during rest and exercise [140]. This study used a 3 T magnet combined with a custom-made surface coil and a pCASL pulse sequence. Using this ASL setup and measuring four young healthy volunteers, it was found that, compared to the resting state (60 ± 5 beats per minute and arterial pressure of 78 ± 5 mmHg), the retinal blood perfusion increased by 25% ± 7% and ocular perfusion pressure increased by 25% ± 6% in the exercise mode.

The same authors investigate the patients suffering from retinitis pigmentosa with similar ASL implementation [141]. The authors measured basal blood flow of 142 ± 16 mL/100 mL/min (or 1.14 ± 0.13 *μ*L/mm^2^/min) in the posterior retinal-choroid in the control group and 70 ± 19 mL/100 mL/min (or 0.56 ± 0.15 *μ*L/mm^2^/min) in the retinitis pigmentosa group. This revealed a significant reduction of blood perfusion in patients compared to controls.

A recent study looked at the effect of aging on choroidal blood circulation, using pCASL and 3 T magnet, scanning 17 normal subjects (24–68 years old) [142]. This study found that choroidal blood flow was negatively correlated with age, declining 2.7 mL/100 mL/min each year, while it was not correlated with perfusion pressure, arterial pressure, or intraocular pressure. This age dependence of choroidal perfusion was only observed in central retina, but not at its periphery.

## 5. Conclusion

**Figure 4:**
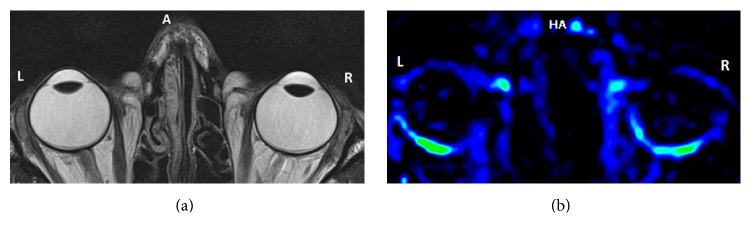
(a) High resolution T2 weighted anatomical image, used to locate the tissue of interest (i.e., the retina) in order to apply the ASL sequence. (b) The perfusion-weighted image calculated from the application of 2D ASL sequence. The choroid layer in the back of the eye is clearly highlighted, showing the detection of blood perfusion into this vasculature bed.

ASL is a versatile and noninvasive imaging technique that is used to estimate tissue perfusion without the use of endogenous tracers. Although the main application of ASL remains the measurement of cerebral blood perfusion, this technique is gradually being adopted in other organs as well. Owing to the nature of the tissues, noninvasive techniques are required to assess pathologies of the eye. While other MRI-based techniques such as T1 and T2 weighted imaging [143], diffusion tensor imaging [144, 145], and contrast-enhanced imaging [146–148] have been used to look at the dynamic physiology of the ocular tissue, ASL shows promise as a technique to assess pathologies of the retina. Here at the University of Auckland, we are implementing new pCASL modalities in our Siemens SKYRA 3 T magnet. While we are still optimizing our approach, our preliminary data are promising (Figure 4).

We are aiming to study the blood perfusion of the retina in healthy and diseased subjects, by using our optimized ASL routine in the very near future. This review article sets the scene for our upcoming research articles, presenting novel research into ocular perfusion.
